# Epiphyseal Fracture of the Coracoid Process Occurring at the Conjoined Tendon Origin

**DOI:** 10.1155/2011/329745

**Published:** 2011-12-20

**Authors:** Kenjiro Nakama, Masafumi Gotoh, Yasuhiro Mitsui, Takahiro Okawa, Fujio Higuchi, Kensei Nagata

**Affiliations:** ^1^Department of Orthopedic Surgery, Kurume University Medical Center, 155-1 Kokubu-machi, Kurume, Fukuoka 839-0863, Japan; ^2^Department of Orthopedic Surgery, Kurume University, 67 Asahi-machi, Kurume, Fukuoka 830-011, Japan

## Abstract

Fracture of the coracoid process is uncommon, and most previous studies have reported this fracture occurring in association with direct trauma to the shoulder or transmission of force from the upper arm or elbow (Ada and Miller 1991, Benton and Nelson 1971, Eyres et al. 1995). We present a case in which epiphyseal fracture occurred at the origin of the conjoined tendon following excessive muscle contraction. We believe this represents the first description of such a method of injury.

## 1. Introduction

**Figure 1:**
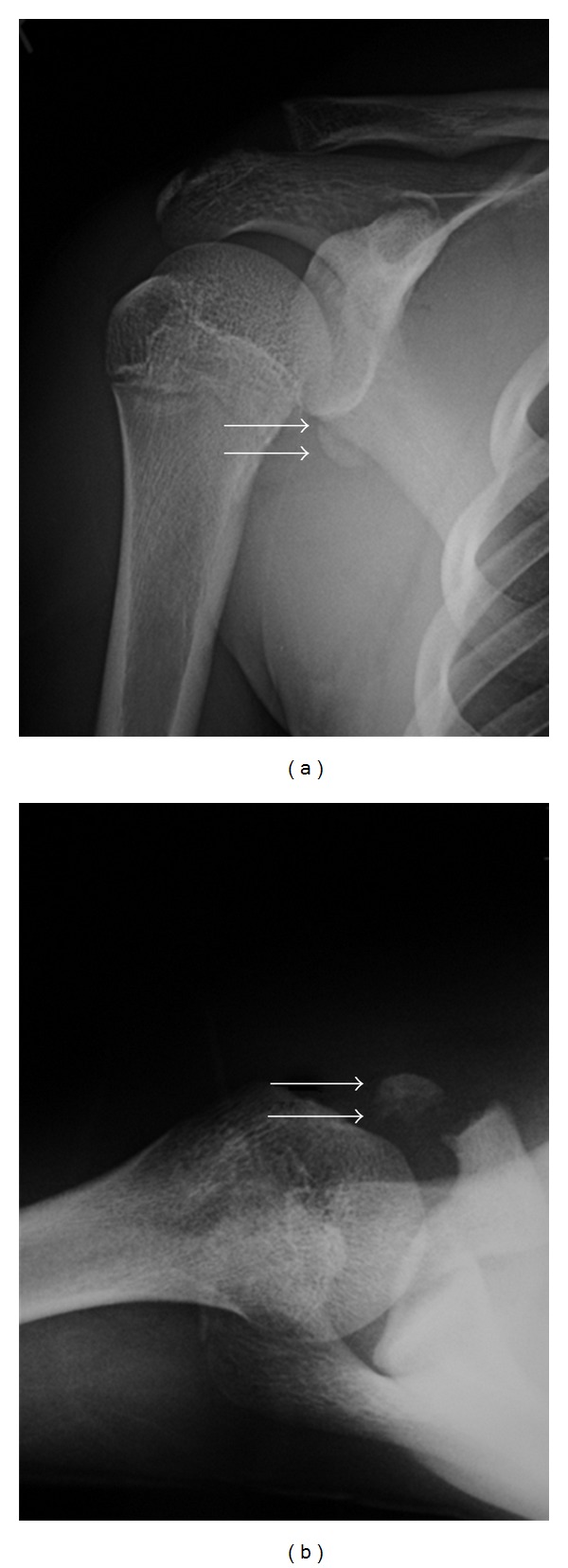
Plain radiographs showing epiphyseal fracture at the origin of the conjoined tendon on the coracoid process. White arrows indicate displaced fragment with coracobrachialis. (a) Anteroposterior view. (b) Axillary view.

**Figure 2:**
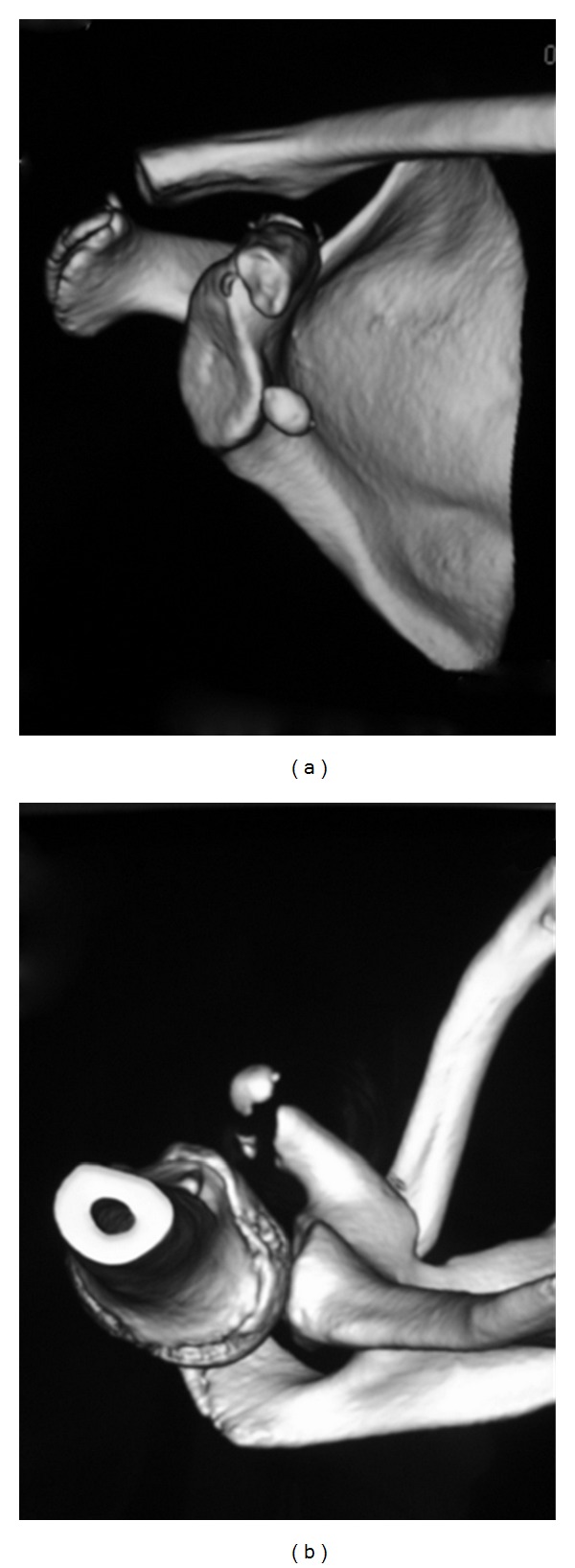
Computed tomography showing two bone fragments resulting from epiphyseal fracture at the origin of the conjoined tendon on the coracoid process. (a) Anteroposterior view. (b) Axillary view.

A 16-year-old boy suffered right-shoulder injury after performing a flying rings routine in a gymnastics competition. He visited a local doctor and was referred to our hospital. On examination, he showed tenderness with mild swelling at the anterior aspect of the shoulder. Range of motion in the shoulder was restricted by pain. Anteroposterior and axillary views of the right-shoulder demonstrated fracture at the top of the coracoid process with normal acromioclavicular joints (Figures 1(a) and 1(b)). Computed tomography revealed displaced and minimally displaced fragments (Figures 2(a) and 2(b)). Operative reduction and internal fixation were subsequently performed.

**Figure 3:**
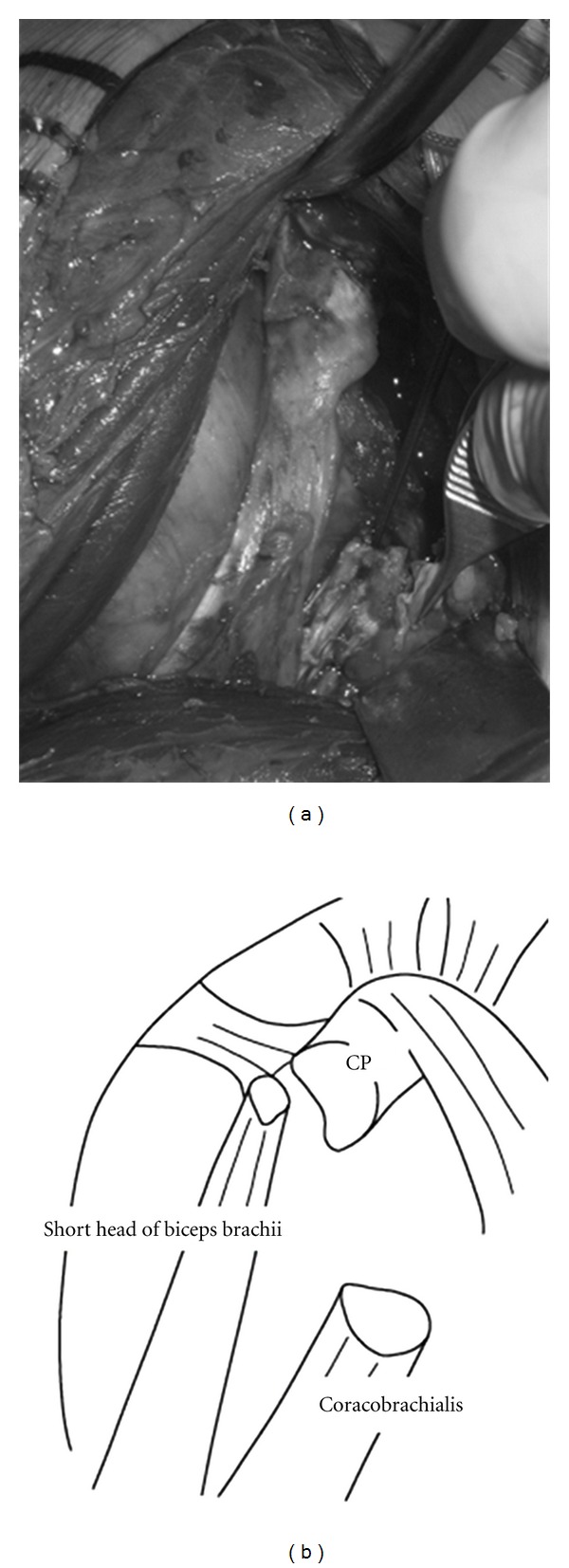
The large fracture fragment with the coracobrachialis had been displaced inferiorly, but the small fragment with part of the short head of biceps brachii had largely maintained its anatomical position; a majority of the origin of the short head of biceps brachii at the coracoid process was retained.

**Figure 4:**
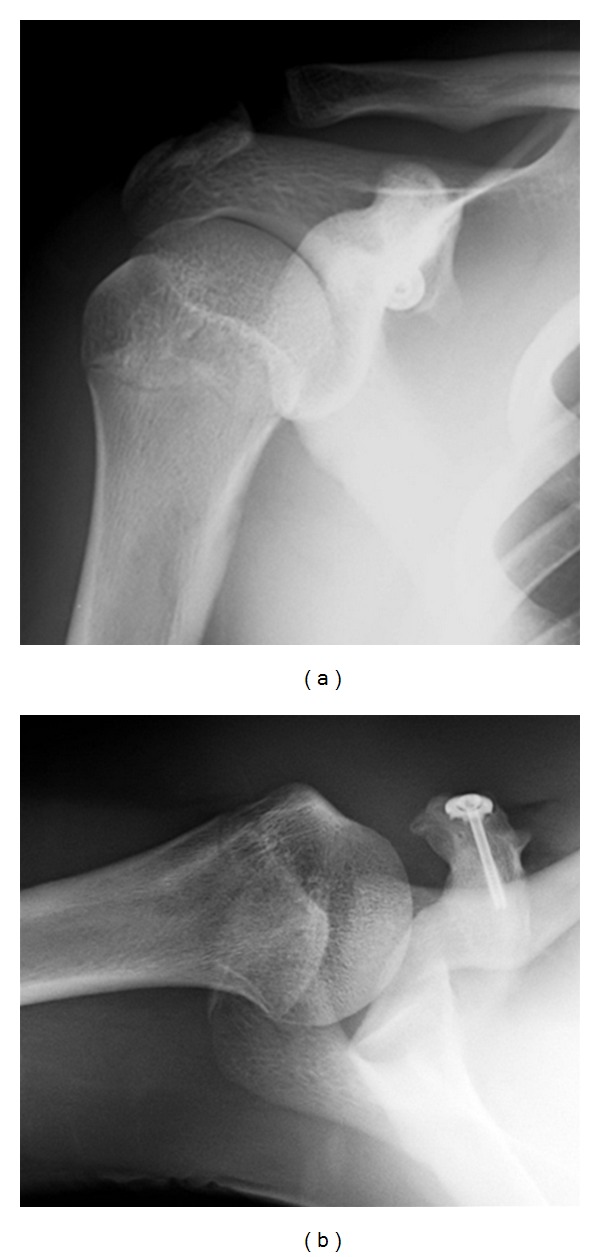
Plain radiographs at 2 years postoperatively. (a) Anteroposterior view. (b) Axillary view.

Using a deltopectoral approach, the coracoid process was exposed. The large fracture fragment with the coracobrachialis had been displaced inferiorly, but the small fragment with part of the short head of biceps brachii had largely maintained its anatomical position; a majority of the origin of the short head of biceps brachii at the coracoid process was retained (Figures 3(a) and 3(b)). The fragment with the coracobrachialis was reduced to the anatomical position and fixed using a cannulated cancellous screw with a washer. The fragment with the short head of the biceps tendon was sutured to surrounding soft tissue. Postoperatively, the shoulder was immobilized using a broad arm sling. Active range of motion was allowed to be started from 3 weeks postoperatively. As of 2 years postoperatively, the patient was pain-free with normal range of motion in the right-shoulder and had returned to his previous sports. At this time, plain radiography demonstrated completed consolidation at the fracture site (Figures 4(a) and 4(b)).

## 2. Discussion

Isolated fractures of the coracoid process are uncommon, representing about 2–5% of all scapular fractures [1]. Most fractures occur at the base of the coracoid process, in association with acromioclavicular dislocation or anterior shoulder dislocation [2, 3]. Fracture of the coracoid process proximal to the epiphysis, apophyseal, or epiphyseal fractures are more uncommon [4–6]. The present case was classified as epiphyseal fracture of the coracoid process, although the fracture pattern was unique, consisting of a displaced fragment with the coracobrachialis and minimally displaced fragment with the short head of biceps brachii. To the best of our knowledge, no such case has previously been reported.

Treatment of an isolated coracoid process fracture depends on the location and displacement of the fracture [1, 3]. In the present case, surgical reduction and fixation were chosen, as (1) the larger fragment of the coracoid had been displaced considerably by contraction of the coracobrachialis muscle and (2) the patient, as a young, top-level athlete, required muscular strength for flying rings routines in competitive gymnastics.

The pathomechanics of the present case may be of great importance. The epiphyseal plate at the point of attachment to the conjoined tendon is assumed to be weaker than after closure [7]. Furthermore, the specific biomechanical forces encountered in flying rings (more forceful muscle contraction in the coracobrachialis than in the short head of biceps brachii) may have developed to the conjoint tendon, consequently generating epiphyseal fracture at the origin of the conjoined tendon in the unique pattern of injury described above.

## 3. Conclusion

The exact pattern of the epiphyseal fracture in this case was unique, consisting of a displaced fragment with the coracobrachialis and minimally displaced fragment with the short head of biceps brachii. In the present report, surgical intervention was required and prognosis was good. This case remains rare, but surgeons should be able to recognize the fracture pattern as seen in the case.
